# Early Clinical Outcomes in Patients of Carcinoma Cervix Treated With Volumetric Modulated Arc Therapy

**DOI:** 10.7759/cureus.46798

**Published:** 2023-10-10

**Authors:** Sri Harsha Kombathula, Pandjatcharam Jagadesan, Mourougan Sinnatamby, Abhilash Menon, Durga Harika Kannikanti, Chandramouli R, John M Mathew

**Affiliations:** 1 Clinical Oncology, East Suffolk and North Essex National Health Service (NHS) Foundation Trust, Essex, GBR; 2 Radiation Oncology, Jawaharlal Institute of Postgraduate Medical Education and Research (JIPMER), Puducherry, IND; 3 Medical Oncology, Malabar Cancer Centre, Thalassery, IND; 4 Radiation Oncology, Krishna Cancer Institute, Cuddalore, IND; 5 Radiation Oncology, Princess Margaret Cancer Centre, Toronto, CAN

**Keywords:** response assessment, clinical outcome, disease free survival, overall survival, toxicity, cancer cervix, volumetric modulated arc therapy, radiotherapy

## Abstract

Objective

Carcinoma cervix is one of the major cancers affecting Indian women. Concurrent chemo-radiotherapy is the standard of care in the treatment of carcinoma cervix. We aimed to study the outcomes and toxicity profile of volumetric modulated arc therapy (VMAT), an advanced modality of radiation delivery when used to treat patients with carcinoma cervix.

Materials and methods

Patients of carcinoma cervix belonging to FIGO (The International Federation of Gynecology and Obstetrics) stages IB2- IVA were recruited into the study. The patients were treated with VMAT to an EBRT (External Beam Radiation Therapy) dose of 50.4Gy in 28 fractions, which was followed by a brachytherapy schedule of 8Gy for each fraction to point A for three fractions. Toxicities were monitored weekly during the course of treatment and thereafter at every follow-up visit. A response assessment CECT (Contrast Enhanced Computed Tomography) scan was done three months after treatment and the response was recorded using RECIST (Response Evaluation Criteria In Solid Tumors) criteria.

Results

Sixty-four patients were available for analysis and most of the patients belonged to stage IIB (50.3%) followed by stage IIIB (28.5%). The complete response rate was 90.6% at three months and at a median follow-up of 12 months, the overall survival was 100% and disease-free survival was 89.1%. An analysis of clinically significant toxicities (grade 2 or worse) showed that diarrhea was the most common (20.3%) followed by proctitis (14%) and anemia (9.3%).

Conclusion

The results of the study established that volumetric modulated arc therapy is an acceptable modality of treatment of carcinoma cervix with an attractive toxicity profile. However, longer follow-ups will provide valuable information regarding the long-term disease control and late toxicities of the treatment.

## Introduction

Though the incidence of cervical cancer is showing a downward slope globally, it still represents a major problem in developing nations. GLOBOCAN 2020 data shows that 96,922 new cases of cervical cancer were registered during the year 2018 in India making it the second leading cancer in Indian women [1]. The incidence of cervical cancer in the Indian population peaks at 55-59 years of age [2]. The lack of awareness among the Indian population regarding screening and the disease is reflected in the majority of patients presenting in the advanced stage (FIGO IIB-IV) [3,4].

Concurrent chemoradiotherapy (CCRT) followed by brachytherapy is the standard of care and has bestowed upon the patients an overall survival (OS) and a progression-free survival (PFS) advantage. A greater incidence of worse-grade toxicities accompanied this improvement in survival and disease control [5].

External beam radiation therapy (EBRT) delivery can be done by various techniques. Owing to the advancements in technology, we are currently moving towards an era of conformal radiotherapy where efforts are directed towards decreasing the dose received by the normal tissues termed organ at risk (OAR) without compromising the dose received by the target tissues. This brings about a gain in the therapeutic ratio (ratio of tumor control probability to normal tissue complication probability).

Volumetric modulated arc therapy (VMAT) is a type of Intensity Modulated Radiotherapy (IMRT) that combines intensity modulation of one to two arcs of rotating beams with variation in dose rate with gantry angle.

With this context, studies have been conducted using IMRT to treat carcinoma cervix and have shown encouraging results. This study aims to establish the efficacy and safety of VMAT in carcinoma cervix which has been evaluated in a prospective setting only in a few studies so far and to contribute to the limited available data.

## Materials and methods

This single-arm prospective trial was approved by the Institute Ethics Committee (Registration number-ECR/342/Inst/PY/2013/RR-19) at Jawaharlal Institute of Postgraduate Medical Education & Research (JIPMER). This trial is registered in the Clinical Trials Registry-India (CTRI) database with the registration number CTRI/2017/11/010438. Written informed consent was taken from the patients. The patients were explained about the details of the treatment, the expected outcomes, and the side effects based on the available data.

Patients with biopsy-proven squamous or adenocarcinoma of the cervix belonging to FIGO Stages IB2 to IVA attending our center were recruited for the study. Patients with any of the following factors like an Eastern Cooperative Oncology Group (ECOG) Performance Status >2, poor renal function (creatinine Clearance<40 ml/min), previous pelvic surgery or irradiation, and second malignancy were excluded. The pre-treatment evaluation comprised a complete hemogram, renal function tests, liver function tests, and contrast-enhanced computerized tomography (CECT) of the thorax, abdomen, and pelvis. Cystoscopy and proctoscopy were performed to confirm radiological evidence of bladder or rectal wall involvement by the growth.

Simulation and target delineation

The patient was simulated in a supine position and immobilized using a four-clamp thermoplastic cast. Bladder protocol (500ml water intake after emptying bladder and half-hour before simulation scan) was utilized with the intent to spare small bowel from unintended radiation and to keep the volume of the urinary bladder constant during External Beam Radiotherapy by VMAT (Volumetric Modulated Arc Therapy). A computerized tomography scan was done with a 3 mm slice thickness after the administration of intravenous iohexol with images captured from above the diaphragm to the level of mid-thigh.

Clinical Target Volume(CTV) was delineated in each of the cases in accordance with the guidelines put forth by Toita et al [6]. CTV included the uterus, cervix, vagina, and regional lymph nodes. The OARs (Organs At Risk) were contoured according to the guidelines of the Radiation Therapy Oncology Group (RTOG) [7]. 

Planning and execution

The planning was done on the treatment planning system (TPS) Eclipse version 10.0 (Varian Medical Systems, Palo Alto, CA). Two rotational arcs 0 to 179 and 179 to 0 with 6 MV X-ray beams were used to generate the dosimetry of the treatment volumes and OARs. The dose was calculated according to the planning system, Eclipse version 10.0, using an Anisotropic Analytical Algorithm (AAA) using the inverse planning technique. Dose distributions are analyzed based on dose gradients. Low dose gradient regions are required to meet the acceptance criteria placed on dose difference, and high dose gradient regions are required to meet the acceptance criteria placed on distance‐to‐agreement. The standard pass criteria set was a 3% dose difference and 3 mm distance to agreement for all plans under this study. All approved treatment plans were verified in the Varian Clinac iX using an electronic portal imaging device (EPID). A mandatory patient-specific quality assurance program was used to facilitate the clinical implementation of all plans. The Varian Eclipse treatment planning system Eclipse version 10.0 generated fluence was compared with the actual fluence generated by the treatment unit using a validated portal dosimetry software version 10.0. The gamma criteria set were 3 mm (DTA or Distance To Agreement) and 3% (DD- Dose Difference) for global and local normalisation. Gamma evaluation was performed on all the treatment plans, and passing rates were more than 95% in all cases. All patients showed agreement between measurements and calculations.

A dose of 50.4 Gy in 28 fractions was prescribed to the planning target volume (PTV) at the rate of 1.8 Gy/fraction. Patients with para-aortic nodal involvement were treated with para-aortic nodal irradiation (45 Gy in 25 fractions). We treated pelvic and paraortic lymph nodes sequentially. We have included the hypodense areas of the bone visible on a simulation computerized tomography scan as the bone marrow- one of the organs at risk. Constraints for planning were set according to Quantitative Analyses of Normal Tissue Effects in the Clinic (QUANTEC) limits [8]. The dose constraints to the marrow were set at V10<90% and V20<75%. Radiation was delivered five days a week on Clinac iXTM (VARIAN). Cone Beam Computerized Tomography was used to verify the treatment setup for the first three consecutive days and weekly thereafter. Priority was given to the PTV at the level of gross tumor volume (GTV) while matching. The constraints with respect to the Bladder of V10 were achieved by only 19 (29.7%) patients whereas the constraint of V20 was achievable in 46 (71.9%) patients. The patients were reviewed once weekly with routine blood investigations. Toxicities if any were recorded and treated accordingly during the course of treatment. The toxicities were graded by the physician using Common Terminology Criteria for Adverse Events (CTCAE) version 4 [9]. The worst grade toxicity noted in each patient was used for analysis. The patients received weekly cisplatin at a dose of 40 mg/m^2^. If the absolute dose for any patient exceeded 60 mg, the dose was split over two days. The maximum number of chemotherapy cycles received was five. The patient’s weight was also monitored throughout the course of EBRT. Patients who had lost significant weight resulting in more than 0.5 cm gap between the cast and the skin were re-simulated and treated with a new plan to account for the change in relative anatomy. Five patients alone required resimulation and replanning due to significant weight loss.

Following the completion of external beam radiotherapy (EBRT), three sessions of high dose rate brachytherapy (8 Gy each to point A) based on a custom institutional protocol (CT image-based dose prescription to point A with geometric optimization to reduce doses to bladder and rectum). Sample Dosimetric data to the rectum and bladder for all three brachytherapy sessions for a patient were as follows. D2cc- Dose received by the 2 cc of the volume of the critical structure receiving the maximum dose. V2- Volume of the critical structure receiving dose more than the ICRU reference point dose. D2cc Bladder- First fraction= 5.1 Gy, Second fraction= 4.3 Gy, Third fraction= 5.3 Gy. D2cc Rectum- First fraction= 4.2 Gy, Second fraction= 3.9 Gy, Third fraction= 3.8 Gy. V2 Bladder- First fraction= 4.5 cc, Second fraction= 3 cc, Third fraction= 5 cc. V2 Rectum- First fraction= 14 cc, Second fraction= 13 cc, Third fraction= 11 cc.

Post-treatment follow up

The patients were evaluated for disease status and toxicities monthly for six months after completion of radiotherapy and bi-monthly for up to one year. Assessment of disease response to treatment was done with a CECT scan (at the third and sixth month after treatment) according to the Response Evaluation Criteria In Solid Tumors (RECIST) criteria RECIST Version 1.1 [10]. The patients with the local residual disease were observed at close follow-ups till three months after treatment with expected further disease regression. At the end of three months, a CECT scan was instituted and if a residual was seen, the patient was considered to have failed treatment if histologically proven and those that failed to show histologic proof were followed up closely for three more months, and imaging was repeated. If the patient had clinical progression of the disease within three months after treatment, she was considered for salvage modalities.

Statistical analysis

The continuous variables such as age, biochemical parameters, and hematological parameters were expressed as mean with standard deviation or median with range. Distribution of variables on categorical variables such as clinical characteristics, treatment profile, histopathological profile, and radiation characteristics, were expressed as frequency and percentages. The association of loco-regional tumor response between the categorical variables mentioned above was carried out by using Chi-square or Fisher's exact test. Comparison of the continuous variables in relation to treatment response or selected toxicities were carried out using independent t-test or Mann-Whitney test or one-way ANOVA/Kruskal-Wallis test, whichever is appropriate based on the number of responses or toxicity categories (two or more than two) and distribution of data. The independent factors associated with the toxicity grades were explored using binary logistic regression analysis or multi-nominal logistic regression analysis. All statistical analyses were carried out at a 5% level of significance and p-values <0.05 were considered significant.

## Results

The median age was 50 years (range 35 to 68 years). Sixty-four patients had completed the intended treatment course with a post-treatment follow-up of up to six months. The majority (71.8%) of the patients presented with complaints of vaginal bleeding. White discharge per vaginum and abdominal pain were the other presenting symptoms.

The most common stage of presentation was FIGO stage IIB which was the case in 35 cases (54.6%). Fifty-two had no medical comorbidities. Most (93.8%) of the patients had biopsy reports showing squamous cell carcinoma. Obturator nodes were the most common nodes involved, followed by external iliac, common iliac, internal iliac, and para-aortic nodes in the order of reducing proportion. There were no abnormal inguinal nodes. The median gross tumor volume was 113 cc (range 27.8 to 254 cc). Chemotherapy dose was modified in 12 patients with creatinine clearance between 40 and 60 mg/ml. The rest of the patients had a creatinine clearance of greater than 60 mg/ml throughout the radiotherapy course. 21.4% of the cases had anemia at baseline and all of them received packed red blood cell transfusions to improve their hemoglobin status. 73.4% of the patients had received at least 200 mg/m^2^ of cumulative cisplatin dose. All patients tested negative for viral markers. The baseline patient characteristics are listed in the table below (Table 1).

**Table 1:** Baseline characteristics

Characteristics		Number of Patients- n(Percentage- %) {64 patients= 100%}
Age	30-40 years	6(9.4%)
	41-50 years	28(43.8%)
	51-60 years	23(35.9%)
	61-70 years	7(10.9%)
FIGO Stage	IB2 stage	2(3.1%)
	IIA stage	2(3.1%)
	IIB stage	35(54.7%)
	IIIA stage	2(3.1%)
	IIIB stage	21(32.9%)
	IVA stage	2(3.1%)
Comorbidities	Hypertension	7(10.9%)
	Diabetes mellitus	6(9.3%)
	Coronary artery disease	1(1.6%)
	RHD- Rheumatic heart disease	1(1.6%)
	Dermatomyositis	1(1.6%)
Histology	Squamous(keratinizing) cell carcinoma	14(21.8%)
	Squamous(non-keratinizing) cell carcinoma	46(71.8%)
	Adenocarcinoma	4(6.4%)
Bleeding	Present	46(71.8%)
	Absent	18(28.2%)
Nodal involvement	No nodes	22(34.4%)
	Obturator nodes	42(65.6%)
	External iliac nodes	20(31.2%)
	Common iliac nodes	8(12.5%)
	Internal iliac nodes	5(7.8%)
	Para-aortic nodes	2(3.1%)
Anemia( Hemoglobin <10 g/dl - grams/decilitre)	Present	13(20.4%)
	Absent	51(79.6%)

Tumor response rate

**Figure 1:**
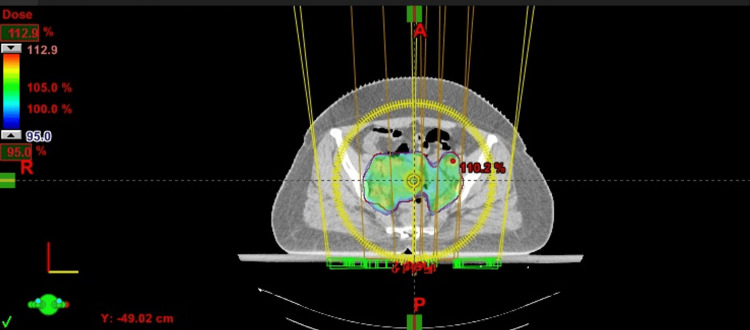
Axial view of dose color wash from a sample plan

**Figure 2:**
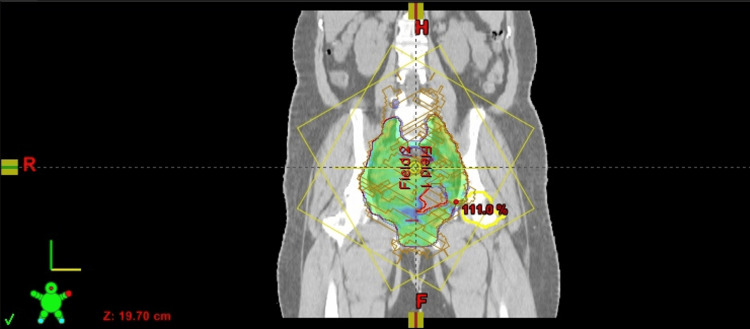
Coronal view of dose color wash from the sample plan

**Figure 3:**
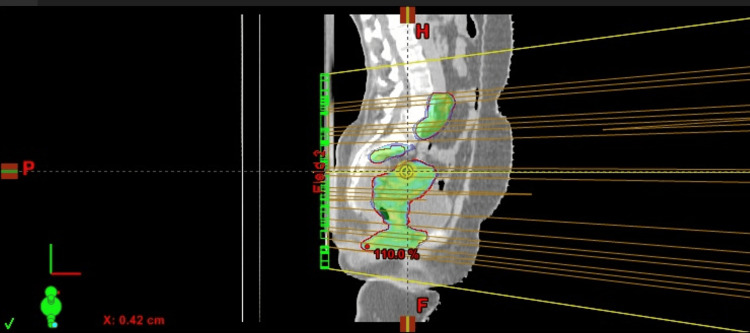
Sagittal view of dose color wash from the sample plan

A sample dose distribution along with the dose-volume histogram is shown in the figure below (Figures 1-3). All the patients were amenable to intracavitary brachytherapy and none had to be treated with interstitial needles. The loco-regional disease control was assessed using Response Evaluation Criteria in Solid Tumors (RECIST) version 1.1. The response assessment CECT was taken at three months and six months after the completion of treatment, at three months, six (9.3%) had treatment failure of which, four (6.2%) were partial responders and progressive disease was noted in two (3.1%) patients. At six months, there were no further relapses. At a median follow-up of 12 months, the overall survival was 100% and the disease-free survival was 89.1%. Seven patients had disease relapse of which two had local only, three had nodal only, one had local and nodal, and one patient developed both nodal and distant relapse to the lungs. PTV coverage (V95%) was similar in the complete and non-complete responders (mean PTV V95% was 96.5% vs 97.2% in the complete and non-complete responders respectively; p=0.059).

The analysis of host, disease, and treatment-related factors revealed that none of them were significantly associated with disease response to the concurrent chemo-radiotherapy regimen as given in the following table (Table 2).

**Table 2:** Association of disease response to various factors GTV T: Gross Tumour Volume in cervix with extensions, if any, to uterus, vagina, parametrium, and adnexa; cc: Cubic centimetre; mg/m^2^:milligrams per metre square; Sixty-four patients: 100%.

Factors	Complete Responders	Non-Complete Responders	p-value
Mean age	47 years	51 years	0.32
Comorbidities present- number of patients- n (percentage- %)	11 (17.2%)	1 (1.6%)	0.57
Histology			0.71
Squamous Carcinoma- number of patients-n(percentage- %)	54 (84.5%)	6 (9.3%)	
Adenocarcinoma- number of patients- n(percentage- %)	4 (6.2%)	0 (0%)	
Mean GTV T Volume	96 cc	110 cc	0.25
Lymphadenopathy- number of patients-n (percentage %)	45(70.3%)	5(7.8%)	0.73
Mean treatment duration (Range)	69(54-108) days	93(52-120) days	0.24
Cumulative chemotherapy dose (Mean)	156 mg/m2	180 mg/m2	0.38

Toxicity profile

No patient had suffered grade IV toxicity of any kind in the study. Toxicity of grade II or worse is considered clinically significant. The most commonly endured non-hematologic acute toxicity was diarrhea followed by proctitis. The most common hematologic toxicity was anemia. Only one patient suffered from grade 3 neutropenia during the treatment course which was managed conservatively. No patient had thrombocytopenia of any grade in the study. During follow-up, the incidence of toxicities was significantly reduced and the only significant toxicities noted were proctitis in 7.8% and cystitis in 1.5% of the sample. Weight loss was noticed in 31 (48.4%) patients during the course of treatment. None of the patients were affected by significant neuropathy/nephropathy. Of the patients that had lost weight, the mean weight loss was 3 Kg. None of the patients lost more than 10% of their baseline body weight. The following table encapsulates the significant toxicities observed in the study (Table 3).

**Table 3:** Toxicity profile CTCAE V4: Common Terminology Criteria for Adverse Events (CTCAE) Version 4.

Acute Toxicity (>/= Grade 2 CTCAE V4)	Number of patients- n (Percentage- %)
Dermatitis	3 (4.8%)
Diarrhea	13 (20.3%)
Vomiting	4 (6.4%)
Proctitis	9 (14.1%)
Cystitis	1 (1.6%)
Anemia	6 (9.4%)
Neutropenia	1 (1.6%)
Thrombocytopenia	0 (0%)

## Discussion

Carcinoma cervix is the second most common cancer afflicting Indian women. This prospective study aimed to assess the toxicities and disease control of VMAT in them. As had been previously mentioned, most of the patients recruited in the study were diagnosed with locally advanced disease. IMRT has been proven to achieve similar disease control rates and better toxicity profiles, especially in terms of hematologic, gastrointestinal, and genitourinary systems [11,12].

In the present series, excellent disease response (90.6% Complete Response by RECIST version 1.1) was achieved even in a mostly locally advanced disease group at three months. At the same time point, 6.2% achieved a partial response, and only 3.1% had disease progression. The disease control rates at various time points of follow-up in similar trials using IMRT/VMAT range from 60% to 93.5% [12-21]. Given the lack of a significant quantity of data showing the outcomes of VMAT, we had to draw a comparison with the clubbed data of both IMRT and VMAT. None of the patients with local-only disease could be salvaged with exenteration in view of extensive disease and poor performance status.

Radiobiologically speaking, a larger dose of radiation results in worse relative toxicity of late-responding tissues thereby resulting in late adverse effects. The rate of accumulation of radiation dose determines the acute adverse effects. Applying the same in the clinical treatment of carcinoma cervix, the brachytherapy regimen influences both the late and acute radiation-induced toxicities whereas the EBRT protocol influences the acute toxicities predominantly. The incidence of significant toxicities in our study is similar to the studies listed in the table below (Table 4).

**Table 4:** Summary of trials evaluating IMRT/VMAT in carcinoma cervix IMRT: Intensity Modulated Radiation Therapy; VMAT: Volumetric Modulated Arc Therapy; GI: Gastrointestinal; GU: Genitourinary; CTCAE V4: Common Terminology Criteria for Adverse Events (CTCAE) version 4; NA: Not Applicable; OS: Overall Survival; PFS: Progression Free Survival; DFS: Disease Free Survival

Trial	Number of patients treated with IMRT/VMAT-n	IMRT/ VMAT	Study type	Stage	Acute GI toxicity (Grade-CTCAE V4)	Acute haematologic toxicity (Grade-CTCAE V4)	Acute GU toxicity (Grade-CTCAE V4)	Outcome
Gandhi et al. [12]	22	IMRT	Prospective	IIB-IIIB	4.5% (>/= Grade 3)	NA	0%(>/= Grade 3)	3-year OS 90.7%
Du et al. [13]	60	IMRT	Retrospective	IIB-IIIB	5.3% (>/= Grade 3)	3.5%(>/= Grade 2)	7.1%(>/= Grade 3)	2-year DFS 60%; 2-year OS 85.7%
Chakraborty et al. [14]	66	VMAT	Retrospective	IB-IIIB	30.3%(>/= Grade 3)	19.7%(>/= Grade 3)	NA	1 year PFS 87%; OS 100%
Mell et al. [15]	83	IMRT	Prospective	IB-IVA	12%(>/= Grade 3)	31.4%(>/= Grade 3)	2.4%(>/= Grade 3)	2-year PFS 78.6%; 2-year OS 90.8%
Hasselle et al. [16]	111	IMRT	Retrospective	IB-IVA	2%(>/= Grade 3)	NA	13%(>/= Grade 2)	3-year DFS 69.2%; 3-year OS 77%
Kidd et al. [17]	135	IMRT	Prospective	IA-IVB	5.1%(>/= Grade 3)	NA	0.7%(>/= Grade 3)	3-year DFS~70%; 3-year OS >90%
Chen et al. [18]	54	IMRT	Prospective	IB-IIA	22%(>/= Grade 3)	34%(>/= Grade 2)	13%(>/= Grade 2)	5-year DFS 71.2%; 5-year OS 64.9%
Wang et al. [19]	373	IMRT	Retrospective	IIB	16.3%(>/= Grade 2)	NA	11.2%(>/= Grade 2)	3-year DFS 82.2%; 3-year OS 87.5%
Yuan et al. [20]	440	VMAT	Retrospective	IA-IVB	2.7%(>/= Grade 3)	Anemia-11.8% Neutropenia- 22.4% (>/= Grade 3)	0(>/= Grade 3)	5-year PFS 71.1%; 5-year OS 74.4%
Lin et al. [21]	398	IMRT	Retrospective	IA-IVB	NA	Anemia-4.5% Neutropenia- 9.1% (>/= Grade 3)	NA	3-year DFS 65.4%; 3-year OS 80.5%

Gandhi et al. in their randomized controlled trial have compared IMRT with conventional radiotherapy and the individuals treated with IMRT had a significantly better toxicity profile in terms of lower grade 2 or worse gastrointestinal and genitourinary effects. The incidence of genitourinary (grade 2 or worse) toxicity in the IMRT arm was 23.8% which is significantly higher than the 1.5% noted in our study. This may be attributed to the fact that a higher proportion of their study population had a stage IIIB disease [12].

In a retrospective review by Chakraborty et al., it was noted that elderly patients (>65 years) also tolerated pelvic radiotherapy by VMAT well with only a non-significant increase in the rate of neutropenia when compared to their younger counterparts. These findings further establish the excellent safety profile of VMAT [14].

The INTERTECC 2 trial which employed functional imaging to delineate and spare bone marrow during radiotherapy prospectively deduced that a reduction in the dose received by bone marrow resulted in a lower incidence of hematologic toxicities. In our study, we delineated the hypodense areas within the bone as marrow which could serve as a substitute for planning organ at risk volume. However, adequate care should be taken to ensure that the PTV coverage is not compromised in an effort to spare the bone marrow [15].

The prospective cohort study by Kidd et al. indicated that IMRT has the potential to improve disease control when compared to the other conventional techniques. They also noted that a PET-CT at three months after treatment is indicative of long-term disease control and survival in patients. Can a CT scan predict the outcomes in the long run is a question that is yet to be answered [17].

VMAT being a highly conformal technique has always been plagued by the uncertainty of geographic miss. This study established that VMAT has a minimal incidence of clinically significant toxicity and is found to have excellent disease response.

Study limitations

The limitations of this study are as follows. Most patients had a longer overall treatment time (OTT) by virtue of sequential external beam therapy and brachytherapy. This could have reduced the incidence and grade of toxicities. Despite a longer OTT, good early disease responses were achieved. The compliance to chemotherapy is also low at only about three-quarters of the sample had received five cycles of chemotherapy. This could have an impact on the toxicity profile of the treatment. Another interesting point is that we haven't boosted involved lymph nodes in the study which has become universally prevalent at present. A lack of volume-based brachytherapy planning though acceptable, can be viewed as a drawback, especially in view of the advanced-stage disease of the sample population. This study has tried to look at the outcomes of VMAT in cervical cancer. However, the follow-up is quite short, especially for cervical cancer.

Though functional imaging is the standard, the hematologic toxicity profile in this study suggests that CT-based contouring of the hypodense areas is a feasible alternative to spare the marrow. We continue to follow up with the study patients to understand the late adverse effects of VMAT and long-term disease control.

## Conclusions

Concurrent chemo-radiation of uterine cervical cancer with Volumetric Modulated Arc Therapy has an excellent toxicity profile and disease response. However, longer follow-ups will provide valuable information regarding the long-term disease control and late toxicities of the treatment. 

To our knowledge, this study is one of the few prospective studies evaluating the disease control and toxicity profile of VMAT in cervical cancers. Another unique nature of our study is the observation that advanced-stage disease formed the majority of the study group patients.

Further studies on dose acceleration and/or escalation, especially to high-risk patients like node-positive and non-squamous histology to improve outcomes may be contemplated with VMAT in view of the excellent toxicity profile.
